# Field cancerization in breast cancer

**DOI:** 10.1002/path.5902

**Published:** 2022-05-03

**Authors:** Emanuela Gadaleta, Graeme J Thorn, Helen Ross‐Adams, Louise J Jones, Claude Chelala

**Affiliations:** ^1^ Centre for Cancer Biomarkers and Biotherapeutics, Barts Cancer Institute Queen Mary University of London London UK; ^2^ Centre for Tumour Biology, Barts Cancer Institute Queen Mary University of London London UK

**Keywords:** breast cancer, field cancerization, cancer‐adjacent tissues, histologically normal

## Abstract

Breast cancer affects one in seven women worldwide during their lifetime. Widespread mammographic screening programs and education campaigns allow for early detection of the disease, often during its asymptomatic phase. Current practice in treatment and recurrence monitoring is based primarily on pathological evaluations but can also encompass genomic evaluations, both of which focus on the primary tumor. Although breast cancer is one of the most studied cancers, patients still recur at a rate of up to 15% within the first 10 years post‐surgery. Local recurrence was originally attributed to tumor cells contaminating histologically normal (HN) tissues beyond the surgical margin, but advances in technology have allowed for the identification of distinct aberrations that exist in the peri‐tumoral tissues themselves. One leading theory to explain this phenomenon is the field cancerization theory. Under this hypothesis, tumors arise from a field of molecularly altered cells that create a permissive environment for malignant evolution, which can occur with or without morphological changes. The traditional histopathology paradigm dictates that molecular alterations are reflected in the tissue phenotype. However, the spectrum of inter‐patient variability of normal breast tissue may obfuscate recognition of a cancerized field during routine diagnostics. In this review, we explore the concept of field cancerization focusing on HN peri‐tumoral tissues: we present the pathological and molecular features of field cancerization within these tissues and discuss how the use of peri‐tumoral tissues can affect research. Our observations suggest that pathological and molecular evaluations could be used synergistically to assess risk and guide the therapeutic management of patients. © 2022 The Authors. *The Journal of Pathology* published by John Wiley & Sons Ltd on behalf of The Pathological Society of Great Britain and Ireland.

## Introduction

Breast cancer is the most common invasive cancer in women worldwide, affecting one in seven women during their lifetime [1]. Widespread mammographic screening programs, enhanced education campaigns, and advances in detection methods and therapeutic regimes allow for the detection of asymptomatic disease, resulting in significant improvements in overall survival [2]. This early detection has led to the increased implementation of breast conservation therapy, which has significantly improved the management of early breast cancer.

Currently, pathological evaluations and genomic tests that focus on the primary tumor are employed to determine therapeutic management and risk of recurrence. Although breast cancer has been studied extensively, patients who undergo breast conservation surgery with post‐operative radiotherapy still have a recurrence rate of between 3% and 15% within 10 years [3].

Originally, it was presumed that local recurrence following surgery was attributable to tumor cells contaminating histologically normal (HN) tissues beyond the surgical margin [4]. However, advances in technology have allowed researchers to better characterize tumors and their associated HN tissues, with several studies reporting aberrations in peri‐tumoral tissues and determining that these tissues provide distinct information beyond that available from tumor. Two main theories have been proposed to explain these observations: the field cancerization theory and the tumor microenvironment theory [5, 6].

The field cancerization theory proposes the creation of a field of molecularly altered cells or an environment with an inherent predisposition to malignant evolution that can occur with or without morphological change [7]; the tumor microenvironment theory suggests that microenvironmental factors are key drivers of tumor initiation and progression. These theories are unlikely to be mutually exclusive but instead co‐exist in an intricate relationship that potentially influences manifested phenotypes [8].

**Figure 1:**
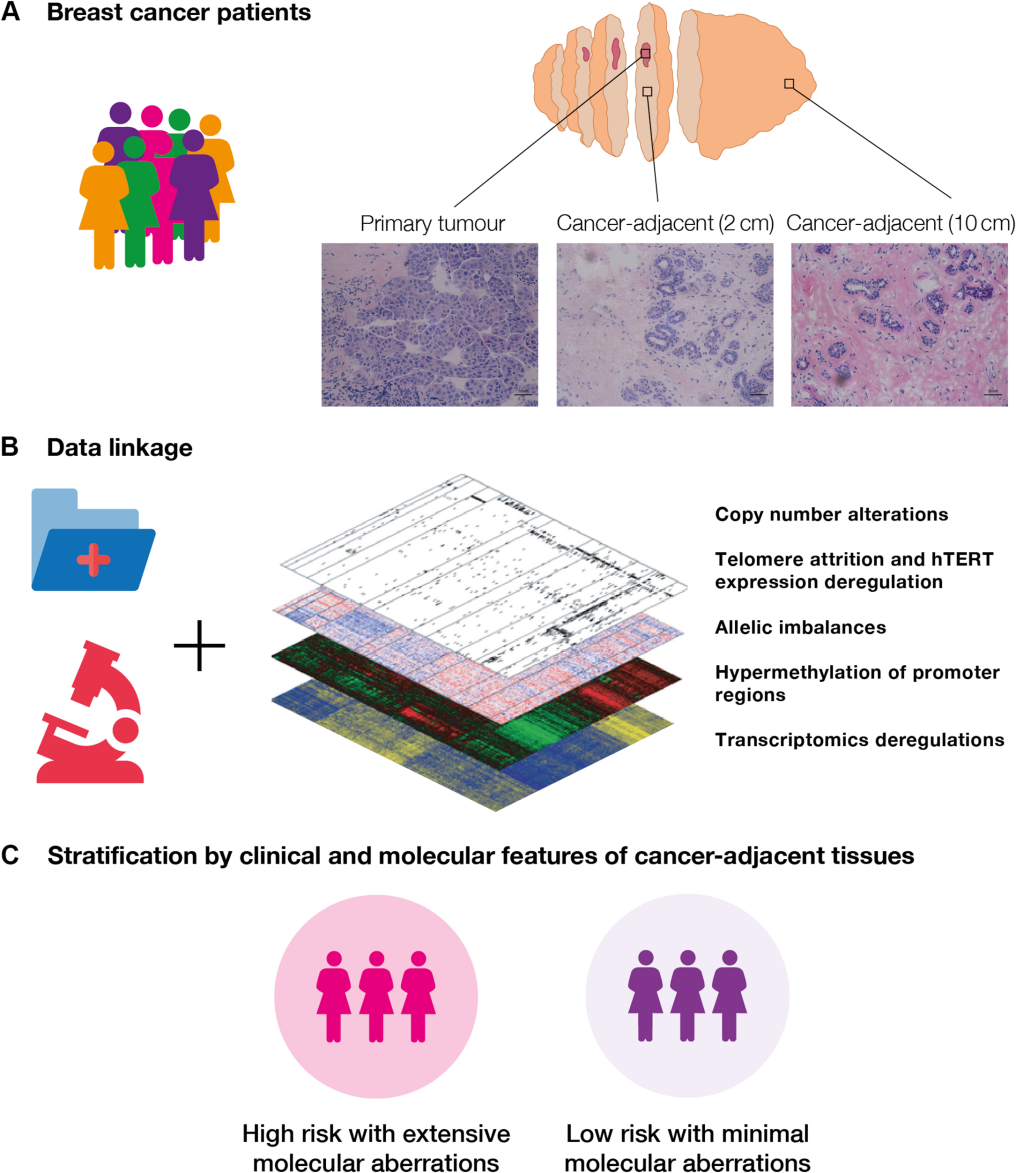
Overview of how healthcare records and individual molecular data could be used to guide stratified care. (A) Patients would be referred to hospital, where a subset would be presented with a breast cancer diagnosis. For each patient, the molecular characteristics of the excised tumor and their associated peri‐tumoral tissues would be recorded in their medical records. (B) Information from both electronic health records (e.g. demographics, pathology, imaging, etc.) and molecular assessments of tumor and surrounding histologically normal tissues (e.g. genomics, transcriptomics, etc.) would be available to clinicians. (C) This information would then be used to stratify patients into clinically significant groups, such as risk of recurrence, or used to help guide the clinical management of patients based on pharmacogenomic vulnerabilities within the residing tissues.

In this review, we explore the concepts of field cancerization in HN tissues. We present the pathologic and molecular characteristics of field cancerization and discuss the implications of using peri‐tumoral tissues in research (Figure 1).

## Development and evolution of field cancerization theories

‘Field cancerization’ was first defined by Slaughter *et al* to describe hyperplastic and atypical epithelia surrounding multifocal oral squamous carcinoma [7]. They attributed the high rate of local recurrence to a progressive change in peri‐tumoral cells rather than the influence of pre‐existing cancer cells. The authors concluded that a preconditioned microenvironmental milieu was created, in which cells underwent irreversible changes to confer tumor growth.

Since this original definition, the concept of field cancerization has been continually adapted to remain abreast of knowledge attained from advances in molecular technologies [9, 10, 11]. In light of evidence from genetic progression models, current definitions support the presupposition that cells within the preneoplastic field accumulate pro‐tumorigenic molecular alterations. However, the tissue itself may not exhibit obvious morphological change as determined by histopathology, meaning that these aberrant cells remain clinically hidden. The criteria that define morphologically normal tissues are being challenged as we begin to understand how ‘normal variation’ may impact cancer risk.

The mechanisms by which the molecularly altered cells develop are not fully understood, but clonal expansion and intra‐epithelial migration of these cells within contiguous epithelial structures have been proposed [11]. It has been suggested that cancerization can be attributed to the divergence, selection, and expansion of one or more clones that ultimately confer a survival advantage over the resident cell population [9].

The ‘etiological field effect’ reassesses the concept of field cancerization by taking a more holistic approach [11]. Here, it is suggested that endogenous and exogenous factors, such as ageing, diet, smoking, and exposure to ultraviolet light, individually and together affect body tissues and contribute to a field of cancer predisposition.

More recently, it has been proposed that field cancerization could be considered in terms of phenotypic traits, rather than underlying specific molecular aberrations. Here, the cancerized field is defined as a collection of cells that have acquired a subset of phenotypic alterations required for malignancy even in the absence of histological alterations [9].

The theories of field cancerization will continue to evolve as studies discern the cellular mechanisms underpinning its aetiology, thus allowing for the demarcation of cancerized fields and elucidation of the properties that define patient outcome and response to therapy.

## Determinants of field cancerization in breast cancer

Technological advances offer the opportunity to gain a deeper understanding of cancer biology and its underlying mechanisms from both a pathological and a molecular aspect. Numerous studies have shown that patches of molecularly altered, cancer‐primed, cells are present in HN tissues surrounding breast tumors and that these cells create an environment with a predisposition to progress to cancer [7, 12, 13, 14, 15, 16, 17, 18, 19, 20]. These studies offer unparalleled insights into the hidden depths of these peri‐tumoral tissues.

### Pathological

The central tenet of histopathology is that molecular alterations are reflected in tissue phenotype, and decades of research have related specific phenotypic changes to disease behaviour. In the breast, there is a well‐defined series of proliferative changes that confer a variable risk for development of breast cancer [21], with the risk being highest for atypical ductal hyperplasia [22].

Molecular studies on atypical ductal hyperplasia have demonstrated some shared genomic alterations with established malignancy [17, 23], which supports its precursor nature and representation of cancerized field. Recognition of these entities and subsequent management of patients based on predicted levels of associated risk are the mainstay of protocol‐driven patient management. However, beyond these well‐recognized benign and atypical lesions, there is almost infinite variation in the histological appearance of ‘normal’ breast tissue as well as more subtle changes not captured in routine diagnostics that may reflect a field cancerization effect. This morphological variation represents the phenotypic manifestation of an individual's genetic and hormonal environment and is increasingly recognized to influence cancer risk.

One such histological variation is manifested by the physiological process of lobular involution [24, 25], whereby there is a reduction in the number of acini in a terminal ductal lobular unit (TDLU) and a reduction in the number of lobules per unit area. The occurrence of lobular involution is most prominent around the peri‐menopausal years but is highly variable between individuals, in terms of both stage of development and extent of development.

The status of lobular involution has been assessed semi‐quantitatively across large patient cohorts and demonstrated that more advanced lobular involution in women who have had a biopsy showing benign breast disease is associated with significantly reduced breast cancer risk [25, 26]. Furthermore, longitudinal assessment of lobular involution in women undergoing multiple biopsies indicates that delayed lobular involution is associated with breast cancer risk. This normal variation in breast histology also appears to modify the risk generated by recognized proliferative lesions. For example, in women with atypical epithelial hyperplasia, patients with no lobular involution in surrounding breast tissue show a significantly higher risk of developing breast cancer compared with those with complete lobular involution, with a relative risk of 7.79 versus 1.49, respectively [27].

Another normal variation in breast tissue composition is reflected in mammographic density. It is well established that high mammographic density, reflecting an increased ratio of fibroglandular elements to adipose tissue, is a major risk factor for breast cancer [28]. Studies suggest increased collagen deposition to be a major contributor to mammographic density [29], with enhanced peri‐ductal collagen alignment, resulting in increased tissue stiffening, potentially mediating the pro‐tumorigenic effects of mammographic density [30].

While mammographic density represents a global reflection of breast cancer risk, the direct impact of collagen density and organization on tumor initiation and progression had been shown in several model systems [31, 32], and roles for stromal activation and epithelial–mesenchymal transition (EMT) have been widely implicated in tumorigenesis [33, 34]. As with lobular involution, subtle features of stromal structure are not captured in routine diagnostic pathology yet may significantly influence subsequent cancer risk.

Linking both mammographic density and lobular involution is the effect of estrogen. Mammographic density is influenced by reproductive and menstrual factors, with pregnancy and the menopause associated with a decrease in density [35]. Similarly, exogenous hormone use also influences breast density [36]. Studies correlating endogenous hormone levels and breast density are inconsistent [37, 38], probably due to multiple confounding factors, though a relationship is supported by the association between some SNPs in the *ESR1* gene and percent mammographic density [39]. Similarly, in both pre‐ and post‐menopausal women, higher circulating levels of estrogen metabolites are associated with significantly higher TDLU counts and reduced lobular involution [40]. These findings support the well‐recognized association between high estrogen levels and breast cancer risk and suggest that at least part of this risk is mediated through modulation of breast phenotype.

The growing digitization of histopathology and advancements in machine learning approaches offer the opportunity to capture and quantitate a greater wealth of information from histological sections, as was shown recently with the application of a convolutional neural network for automated quantitation of lobular involution in breast biopsies [41]. This opens the potential to better assess field cancerization features in apparently normal tissue and improve patient care.

### Molecular

The risk of breast cancer recurrence is determined primarily by histopathological evaluations of the tumor, the results of which are used to guide the therapeutic management of patients. However, genetic, epigenetic, and transcriptomic alterations have been identified in cancer‐adjacent tissues of many epithelial cancers, including head and neck [7, 12], colorectal [13, 42, 43, 44], skin [45, 46], bladder [13, 47, 48, 49], lung [14, 50], prostate [8, 13, 51, 52], ovarian [53, 54], and breast [10, 13, 14, 15, 18, 19, 20, 55, 56, 57, 58, 59, 60, 61, 62, 63, 64, 65, 66, 67, 68]. Moreover, persistent exposure to environmental risk factors (e.g. tobacco carcinogens and diet) and infectious agents (e.g. human papilloma virus and *Helicobacter pylori* infections), and the presence of physiological conditions (e.g. diabetes), can further induce molecular insults in cancer‐adjacent tissues, resulting in an environment primed for tumor development [42, 69, 70, 71].

Evidence suggests that HN tissue resected adjacent to primary tumors can be prognostic [13, 72, 73]. Furthermore, characterization of the spatial landscape of the cancerized field has shown that molecular aberrations exist in morphologically normal tissues excised up to 24 cm from the primary tumor, and that the field itself is composed of distinct profiles of plasticity [55]. These observations suggest that assessing tumors in combination with their surrounding tissues could improve prognostic and therapeutic determinations.

Pan‐cancer studies provide a comprehensive overview of the mechanistic profiles and aberrant pathways shared in peri‐tumoral tissues across cancer cohorts, such as enrichment in immune response/inflammation, metabolism, and cell growth [13, 14, 48]. These large‐scale studies use sequencing and SNP data available from The Cancer Genome Atlas (TCGA). While being an invaluable source of large‐scale sample data generated from an array of platforms (e.g. RNA‐seq, DNA‐seq, methylation arrays, CGH, and SNP arrays, etc.) and sample types (e.g. tissues from tumor, metastasis, and cancer‐adjacent tissues as well as blood‐derived normal), TCGA does not provide information pertaining to the margins of excision, thereby limiting extrapolations of the spatial implications of the cancerized field. Pan‐cancer studies are a robust method to highlight alterations shared across cancer types, but they can lack the granularity offered by cancer‐specific research.

Genomic instability is a major characteristic of most cancers that has also been observed in peri‐tumoral tissues in breast cancer [61, 64, 66]. Defining events of genomic instability include telomere attrition, allelic imbalance (including loss of heterozygosity), promoter hypermethylation, copy number alterations (CNAs), and somatic mutations.

Table 1 presents an overview of key molecular markers reported to be associated with field cancerization in breast cancer.

**Table 1:** Molecular alterations reported to be associated with field cancerization in breast cancer.

Molecular alteration	Summary of observations	Specimens studied	Reference
Genomics: telomere attrition	Telomere attrition was observed in both cancer cells and luminal epithelial cells in HN TDLUs adjacent to cancer	Tumor Matched cancer‐adjacent tissue	Kurabayashi *et al* [74]
Genomics: telomere attrition and unbalanced loci	Telomere attrition and unbalanced loci were observed in HN cancer‐adjacent tissues, with this genomic instability being a function of distance from tumor	Matched HN tissue excised 1 cm from tumor margin (TAHN‐1) Matched HN tissue excised 5 cm from tumor margin (TAHN‐5) Healthy breast tissue from reduction mammoplasty	Heaphy *et al* [10]
Genomics: allelic imbalance	Allelic imbalance in matched HN microdissected breast TDLUs from breast cancer patients and *BRCA1* mutation carriers was three‐fold greater than in the reduction mammoplasty control group	HN microdissected breast TDLUs from sporadic breast cancer patients (precise distance from tumor is indeterminate) HN microdissected breast TDLUs from *BRCA1* gene mutation carriers HN microdissected breast TDLUs from reduction mammoplasty	Larson *et al* [59]
Genomics: allelic imbalance	The mean frequency of allelic imbalance was higher in HN tissue adjacent to the primary cancer (15.4%) relative to distant tissue from the same breast (3.7%)	Tumor (laser‐assisted microdissection) DCIS (laser‐assisted microdissection) Matched disease‐free tissue adjacent to the primary tumor (laser‐assisted microdissection) Matched HN distant tissue (laser‐assisted microdissection)	Ellsworth *et al* [68]
Genomics: loss of heterozygosity	Normal breast epithelial cells obtained from women whose risk of breast cancer had been calculated using the Gail model identified associations between frequency of loss of heterozygosity and patient risk, with a lower risk score of 16.7% reported in patients without loss of heterozygosity, compared with 22.9% if loss of heterozygosity is present	Fine needle aspirations from asymptomatic women with known Gail risk score	Euhus *et al* [63]
Genomics: differential methylation at CpG loci	Significantly more CpG loci were identified as differentially methylated between contralateral‐normal and tumor (63 271 CpG loci *q* < 0.01) than between ipsilateral‐normal and tumor (38 346 CpG loci *q* < 0.01). Furthermore, differential methylation in ipsilateral‐normal relative to contralateral‐normal tissue (9562 CpG loci *p* < 0.01) was also observed	Tumor Matched ipsilateral HN tissue excised ≥3 cm from tumor margin Matched contralateral HN tissue from contralateral breast	Muse *et al* [65]
Genomics: genome‐wide DNA methylation and copy number calls	Identified hypervariable levels of DNA methylation and copy number alterations (CNAs) in normal‐adjacent tissue (relative to tissue from healthy breast samples), which then became further enriched in the matched tumors. Furthermore, changes in DNA methylation in normal cells were more predictive of breast cancer status than their CNV counterparts	Tumor Unmatched cancer‐adjacent tissue excised ≥3 cm from tumor margin Healthy breast from reduction mammoplasty Tumor (TCGA) Matched cancer‐adjacent tissue excised ≥2 cm from tumor margin (TCGA)	Gao *et al* [64]
Genomics: hypermethylation of *RUNX3*	Normal tissue in close proximity to the primary tumor exhibited hypermethylation of *RUNX3*	Tumor Matched HN tissue excised 1 cm from visible tumor boundary (N1) Matched HN tissue excised 2 cm from visible tumor boundary (N2) Matched HN tissue excised 3 cm from visible tumor boundary (N3) Matched HN tissue excised 4 cm from visible tumor boundary (N4) Healthy breast tissue from reduction mammoplasty	Cheng *et al* [75]
Genomics: hypermethylation and allelic imbalance of *RASSF1A* promoter region	Four locations (1, 2, 3, and 4 cm) in HN breast tissue from the affected and contralateral breast of breast cancer patients identified hypermethylation in the ipsilateral samples relative to the contralateral with the effect being more pronounced in the vicinity of tumor	Tumor Matched HN tissue excised 1 cm from tumor margin (TAHN‐1) Matched HN tissue excised 2 cm from tumor margin (TAHN‐2) Matched HN tissue excised 3 cm from tumor margin (TAHN‐3) Matched HN tissue excised 4 cm from tumor margin (TAHN‐4) Healthy breast tissue from reduction mammoplasty	Yan *et al* [61]
Genomics: promoter methylation	*RARβ2* was observed hypermethylated with cancer‐adjacent tissues (32%) relative to unaffected breast (9%). Promoter methylation of *RASSF1A* and *APC* occurred more frequently in breast tissues from unaffected women at high risk for breast cancer than in tissues from women at low/intermediate risk of breast cancer	Tumor (fine needle aspiration) Matched benign tissue (fine needle aspiration). Samples taken from ipsilateral breast in quadrant opposite to cancer, with precise distance from tumor indeterminate Unaffected patients (fine needle aspiration)	Lewis *et al* [19]
Genomics: copy number alterations and loss of heterozygosity	Identification of tissue‐specific CNAs in cancer‐adjacent tissues from solid tumors, including breast invasive carcinoma	Tumor (TCGA) Matched cancer‐adjacent tissue excised ≥2 cm from tumor margin (TCGA)	Jakubek *et al* [48]
Genomics: copy number alterations	Study of 282 females with sporadic breast cancer identifies 108 patients (38.3%) with cancer‐associated CNAs in at least one aberrant cancer‐free breast tissue	Tumor Matched HN tissue (Polish cohort, Bydgoszcz) excised 4–8 cm from tumor margin Matched HN tissue (Swedish cohort, Falun) excised at variable distances from tumor margin Matched HN tissue (Polish cohort, Krakow) excised at variable distances from tumor margin (maximum 15 cm) Matched HN (tissue Polish cohort, Gdansk) excised at variable distances from tumor margin Healthy breast tissue from reduction mammoplasty	Forsberg *et al* [55]
Genomics and transcriptomics: profiling	Breast epithelial samples obtained from ducts leading to breast carcinomas and matched samples from ducts on the opposite side of the nipple. Determined increased mRNA perturbation in proximity to the primary tumor, with these aberrations not being explained by CNAs	Tumor Matched HN tissue taken from duct between tumor and nipple (D1) Matched epithelial sample closest to tumor with some samples exhibiting atypical hyperplasia taken from duct between tumor and nipple (D2) Matched HN control taken from the duct on the other side of the nipple within the same breast (O1)	Abdalla *et al* [66]
Genomics and transcriptomics: profiling	About 40% of HN cancer‐adjacent tissues harbored genomic defects in DNA copy number (10%), sequence, methylation, or in RNA sequence (>40%). These molecular alterations were not associated with significant differences in overall survival	Tumor (TCGA) Matched cancer‐adjacent tissue excised ≥2 cm from tumor margin (TCGA)	Troester *et al* [72]
Transcriptomics and proteomics: profiling	Transcriptomic and proteomic analysis of breast tumors and matched HN tissues resected proximal to (<2 cm) and distant from (5–10 cm) the primary tumor, using tissues from reduction mammoplasties as baseline. Four distinct transcriptomic subtypes are identified within matched normal tissues: immune; matrisome/EMT, non‐coding enriched and metabolic, with the latter associated with poor prognosis (*p* < 0.001, HR 6.1)	Tumor Matched HN tissue resected <2 cm from primary tumor (proximal) Matched HN tissue resected 5–10 cm from primary tumor (distal) Prophylactic mastectomy Healthy breast tissue from reduction mammoplasty Tumor (TCGA) Matched cancer‐adjacent tissue excised ≥2 cm from tumor margin (TCGA)	Gadaleta *et al* [57]
Transcriptomics: profiling	Pan‐cancer study in which profiles from cancer‐adjacent tissues were deemed to represent an intermediate state between tumor and healthy, with both tumor‐associated and unique features observed in cancer‐adjacent tissues.	Tumor (TCGA) Matched cancer‐adjacent excised ≥2 cm from tumor margin (TCGA) Healthy breast from autopsy (GTEx)	Aran *et al* [13]
Transcriptomics: profiling	Pan‐cancer study in which profiles from cancer‐adjacent tissues were found to provide more information about patient survival than tumors	Tumor (TCGA) Matched HN excised ≥2 cm from tumor margin (TCGA)	Huang *et al* [14]
Transcriptomics: molecular subtypes	Transcriptomic analysis of two breast cancer datasets observed intrinsic tumor subtypes to be reflected in HN cancer‐adjacent tissues, with this observation not dependent on distance from primary tumor	Tumor (PWBCS cohort) Matched HN tissue excised <2 cm from tumor margin (PWBCS cohort) Tumor (TCGA) Matched cancer‐adjacent tissue excised ≥2 cm from tumor margin (TCGA) Matched HN tissue excised <2 cm from tumor margin (peri‐tumoral, NBS cohort) Matched HN tissue excised >2 cm from tumor margin (remote, NBS cohort)	Casbas‐Hernandez *et al* [62]
Transcriptomics: profiling	Transcriptomic analyses stratified profiles from HN tissues adjacent to breast cancer or ductal carcinoma *in situ* (DCIS) data into two extratumoral subtypes: *active* and *inactive*, with the *active* subtype significantly associated with overall survival in estrogen receptor (ER)‐positive patients (HR 2.5, *p* = 0.062) and hormone‐treated patients (HR 2.6, *p* = 0.045)	Tumor DCIS Matched HN tissue excised <2 cm from tumor/DCIS margin (peri‐tumoral) Matched HN tissue excised >2 cm from tumor/DCIS margin (remote)	Román‐Pérez *et al* [60]
Transcriptomics: *TERT* gene expression	Normal tissue proximal to breast tumors were found to contain a population of human mammary epithelial cells (HMECs) that expressed human telomerase reverse transcriptase (hTERT) expression levels similar to HMECs within the tumor. hTERT expression decreased in HMECs from HN tissues at increased distance from tumor	Tumor Matched HN tissue excised 1 cm from tumor margin (TAHN‐1) Matched HN tissue excised 3 cm from tumor margin (TAHN‐3) Matched HN tissue excised 5 cm from tumor margin (TAHN‐5) Healthy breast tissue from reduction mammoplasty	Trujillo *et al* [15]
Transcriptomics: profiling	Comparison of the gene expression profiles of microdissected HN epithelium from tissues of patients with breast cancer, at high risk of breast cancer, and from reduction mammoplasty identified defining features within the HN tissues of patients with breast cancer relative to reduction mammoplasty	HN epithelium from breast cancer patients HN epithelium from prophylactic mastectomy HN epithelium from reduction mammoplasty	Graham *et al* [58]
Transcriptomics: profiling	Transcriptomic profiling and immunohistochemistry discerned a signature of differential gene expression that discriminated between paired breast tissues excised at resection margins of 1 and 5 cm from the primary tumor	Tumor Matched HN tissue excised 1 cm from tumor margin (TAHN‐1) Matched HN tissue excised 5 cm from tumor margin (TAHN‐5) Healthy breast tissue from reduction mammoplasty	Trujillo *et al* [73]
Transcriptomics: profiling	Gene expression profiling of normal‐appearing TDLUs of ER‐positive breast cancer patients (*n* = 14) and of reduction mammoplasty (*n* = 15) identified 105 differentially expressed genes. Investigations identified cancer‐associated alterations in the normal‐appearing TDLUs relative to TDLUs from reduction mammoplasty	HN microdissected HN breast TDLUs adjacent to untreated ER‐positive breast cancer HN microdissected HN breast TDLUs from reduction mammoplasty	Tripathi *et al* [76]

#### Genetic and epigenetic aberrations in breast cancer

##### Telomere attrition and allelic imbalance

These have been identified in both tumor and matched cancer‐adjacent tissues [10, 55, 59, 61, 67, 74]. Telomeres in paired HN tissue resected within 1 cm from the tumor margin have been reported to be significantly shorter than in paired peri‐tumoral tissues resected at 5 cm [10]. Similarly, the frequency of allelic imbalance and loss of heterozygosity is reported to decrease with increased distance from tumor [68]. The cancer‐adjacent allelic imbalance profiles revealed substantial concordance with those of tumor, implying clonal evolution [10, 56].

The Gail model is a predictive breast cancer risk assessment algorithm that uses demographic and clinical data to estimate a woman's absolute risk of developing breast cancer within specific time frames (up to the age of 90 years), with a score exceeding 1.67% being defined as high risk [77, 78, 79]. Euhus *et al* conducted a study in which 30 asymptomatic women, whose risk of breast cancer had been calculated using the Gail model (11 with normal cytology and 19 with proliferative cytology), underwent breast epithelial cell sampling via fine‐needle aspirate. Associations between the frequency of loss of heterozygosity and cancer risk were reported, with the patient's lifetime risk score increasing from 16.7% to 22.9% if loss of heterozygosity was present [63].

##### Promoter hypermethylation

Aberrant DNA methylation has been observed in tissues from tumor and matched HN tissues, with the methylomic landscape of cancer‐adjacent tissue being epigenetically distinct from that of tissue extracted from the contralateral breast [65]. This observation is supported by inter‐individual studies in which the cancer‐adjacent tissues are found to be more highly methylated compared with healthy control reduction mammoplasty samples [64].

Promoter region hypermethylation has been observed in both cancer‐adjacent tissues and *in situ* breast carcinomas [18, 19, 61, 64, 75]. Hypermethylation of the *RASSF1A* promoter was observed in both tumor and cancer‐adjacent tissues. Separately, hypermethylation of promoters, including *RASSF1A*, was found to be indicative of increased risk of breast cancer: present in 70% of benign breast tissues from women at high risk (Gail risk index ≥ 2) compared with 29% of women at low/intermediate risk [63].

##### Copy number aberrations (CNAs)

While CNAs have been identified in HN tissues adjacent to cancer, the prevalence and degree of these perturbations in peri‐tumoral tissues are not as extensive as other genomic and transcriptomic events and they do not appear to confer predictive capabilities [55, 61, 64, 66, 72]. Furthermore, cancer‐adjacent tissues primarily exhibit tumor‐associated CNA profiles, with gains in established oncogenes and growth factor receptors, such as *ERBB2*, *EGFR*, and *FGFR1* [48, 55].

##### Mutations

Cancer‐associated somatic mutation profiles appear more frequent relative to CNAs in cancer‐adjacent tissues. Troester *et al* reported 25% of cancer‐adjacent samples in TCGA to have moderate‐to‐high levels of tumor‐like somatic mutations relative to CNAs in 10% in the triplet samples analysed, although the variant allele fraction was low (typically less than 5%), which was consistent with matched tumor cellularity [72]. Similarly, mutation hotspots in specific genes, including *ZNF143*, *ALDOA*, and *LEPROTL1*, have also been shown to reflecte proximity to the primary tumor [66].

The occurrences of genomic instability presented suggest that local peri‐tumoral tissues provide a permissive environment for tumorigenesis and progression, with attenuation of this effect with increased distance from the primary tumor.

#### Transcriptomic aberrations in breast cancer

Cancer‐adjacent tissues are often considered to represent an intermediate state between tumor and healthy breast tissue, with their transcriptomic profiles sharing features of both. However, distinct gene expression characteristics defining HN tissues and their associated subtypes suggest that they represent a distinct entity [13, 15, 57, 58, 60, 62, 72].

Global transcriptomic analyses have linked genes differentially expressed in HN peri‐tumoral tissues to a range of deregulated cancer‐associated pathways, including wound healing, extracellular matrix remodelling, altered metabolism, and EMT [13, 15, 57, 58, 60, 72]. Specific prognostic signatures have consequently been developed [5, 57, 60, 72]. Román‐Pérez *et al* identified distinct *active* and *inactive* transcriptomic subtypes in cancer‐adjacent tissues from estrogen receptor‐positive patients, with the former correlating with poor outcome [60]. This *active* mRNA/miRNA signature was also identified in 40% of matched cancer‐adjacent breast tissues (*n* = 142) from the TCGA breast cohort, with this subtype associated with worse poor 10‐year survival in estrogen receptor‐positive patients [72]. Moreover, analysis of TCGA data revealed that gene expression profiles of peri‐tumoral tissues correlate more closely with clinical outcome measures than their corresponding tumors [14].

Several transcriptomic studies also report that the number and degree of aberrations are dependent on distance from the primary tumor [13, 15, 66], in concordance with findings on genomic instability. However, we identified four distinct transcriptomic subtypes within matched peri‐tumoral tissues excised adjacent to (<2 cm) and distal from (5–10 cm) the primary breast tumor, which were found to be independent of distance from tumor. Most importantly, we found the *metabolic* subtype, characterized by deregulation of mediators involved in metabolic processes, lipid and cholesterol metabolism, and hypoxia‐related events, to be significantly associated with poor prognosis [hazard ratio (HR) 6.1]. We also observed a matrisome/EMT subtype that was enriched in matrisomal elements; however, the small sample size and differing sampling design of the TCGA validation cohort prevented meaningful conclusions as to the clinical implications of this group.

That stromal alterations may contribute to a field cancerization effect is supported by Trujillo *et al*, who analysed HN breast tissue taken 1 and 5 cm from invasive breast cancer and compared the gene expression profile with reduction mammoplasty tissue [73]. They identified a gene expression signature reflecting extracellular matrix remodelling, fibrosis, and EMT, which was more prevalent in tissues closest to the tumor and absent in reduction mammoplasty tissue. Similarly, the *active* miRNA/RNA subtype identified in HN tissues represented processes associated with activated stroma or EMT [60, 72].

Taken together, these studies suggest that the molecular profiles of cancer‐adjacent tissues both reflect those of the tumor and possess distinct intrinsic features. Significant heterogeneity in the cancerized field is observed, with clear clinically relevant molecular events linked directly to prognosis occurring even distal from the primary tumor.

Further study is required to determine the extent to which molecular aberrations contribute to the preconditioned milieu for tumorigenesis. With genomic and transcriptomic events being inherently intertwined, a prognostic tool incorporating a combination of these events will likely provide the greatest predictive value.

## Challenges for research

### Access to well‐annotated specimens from biobanks

Well‐annotated tumor and peri‐tumoral samples with comprehensive clinical data are key to fully understanding the landscape of field cancerization. Here, the role of biobanks is crucial. Historically, these were localized specimen repositories set up for the requirements of specific research projects. As the infrastructures developed, biobanks soon became national and international resources designed to accelerate translational research efforts. Specimens supplied by biobanks are the cornerstone to generating molecular data and in allowing biobanks to evolve away from a narrow specimen‐focused approach towards a data‐driven future. However, for biobanks to reach their full potential, they must provide access to high‐quality clinical samples linked to comprehensive clinical, spatial, and molecular information.

One biobank that has recognized and anticipated the importance of complete annotation and data legacy is the Breast Cancer Now Tissue Bank (BCNTB) [80]. The BCNTB ensures that pathologists and researchers work side‐by‐side to provide well‐annotated specimens from an extensive range of tissues and body fluids. These include tumors and tissues taken at specified distances from margins of resection (adjacent <2 cm and surrounding 5–10 cm), which allow researchers to map the spatial characteristics of the cancerized field. Furthermore, the BCNTB has implemented a data return policy in which researchers are required to return research data generated from projects using BCNTB samples, meaning that each set of specimens gains additional layers of molecular and pathological data. This then becomes available to subsequent researchers, allowing the knowledge to build up incrementally, and is available to query from a dedicated BCNTB Analytics Hub [81].

Biobanks need to provide an active response to the changing needs of researchers and anticipate specimen and data requirements, thus ensuring that these are exploited to their full clinical potential.

### Coding of field cancerization concepts in electronic health records (EHRs)

The ability to access and interpret all information within EHRs is a prerequisite to understand disease processes, and to tailor treatments and health services effectively and safely for patient benefit. NHS England has announced that it will fund an initiative to audit metastatic breast cancer to provide accurate information about patients living with this disease and reform how metastatic breast cancer is currently coded in EHRs [82], which will increase the understanding of recurrence and the patient's clinical journey. While EHRs currently include information pertaining to the status of the resection margins, there are few molecular data available relating to this margin and its surrounding tissues.

Genomics initiatives, such as the 100,000 Genomes Project, allow for a new branch of genomic medicine to serve the NHS, helping to realize the promise of personalized medicine and driving cutting‐edge research. If field cancerization is responsible for recurrence, then limiting sequencing to tumors alone could prove a missed opportunity to elucidate whether the cancerized field arises from tumor influence, or whether it is, itself, an independent entity that drives tumorigenesis and recurrence. Introducing coding concepts, both histological and molecular, for field cancerization into EHRs to complement histological assessments could drive new opportunities for patient‐centered research and information.

### Balancing the benefits and harm of defining a high‐risk cancerized field

Breast cancer screening programs enable the detection of asymptomatic breast cancer, allowing for early intervention and resulting in reductions of up to 20% in absolute mortality in the women screened [83]. However, screening programs exacerbate overdiagnosis, detecting indolent cancers that would not have given rise to clinically relevant disease [83, 84, 85].

Estimates of overdiagnosis vary from near zero to 50%, with these patients subject to unnecessary treatment, increased psychological stress, and potential adverse reactions and complications associated with treatment. Variations in these estimates are dependent on the definition of overdiagnosis applied. While the numerator represents the number of patients overdiagnosed, the denominator varies between studies depending how this value is defined: by screening alone; during the whole screening period; during both the screening and the interval period; or during the screening period and for the remainder of the individual's lifetime [83, 84].

Longitudinal studies, integrating molecular, pathological, and epidemiological data with corresponding patient clinical records, offer researchers the opportunity to characterize molecularly aberrant fields that generate a pro‐tumorigenic environment. They could also help identify patients, in whom these fields are present, who will progress to cancer, which will be essential to minimize overdiagnosis. Furthermore, these investigations also have the potential to provide insights into whether the defining molecular and phenotypic characteristics of a cancerized field categorized as high risk can be modulated by modifications to environmental factors.

### The added value of molecular pathological epidemiology

To better understand the cancerized field and its implication for breast cancer, researchers need to explore beyond the boundaries of traditional methods. Novel lines of evidence point towards the benefits of integrating molecular pathology and epidemiology – termed molecular pathological epidemiology – to enable researchers to link factors such as environmental exposures and host genetics to pathologic features [86, 87, 88, 89, 90, 91].

Endogenous and exogenous factors (exposures) can contribute to specific disease processes and heterogeneity of neoplastic disease: for instance, antibiotic‐induced microbial dysbiosis in the gut is associated with tumor progression [92, 93, 94, 95, 96]. Emerging evidence of the relationship between dysbiosis and breast cancer has uncovered microbial signatures providing type‐specific communities of organisms distinct to each breast cancer type [97, 98]. Differences in breast microbiota DNA profiles from tumors relative to paired cancer‐adjacent tissues and healthy controls have also been reported, with the microbial profile of cancer‐adjacent tissues exhibiting greater similarities with their corresponding tumor than with healthy controls [93, 97, 98].

While it is possible that these studies indicate that dysbiosis influences the environment surrounding a tumor, potentially modulating the risk of breast cancer, the reverse may also be true, in that the cancerized field itself creates a niche favoring differential microbiome composition, which in turn may influence progression of the disease.

The field of molecular pathological epidemiology allows for the examination of complex relationships between a compendium of exposures. For instance, positive associations have been reported between increased body mass index, hip‐to‐waist ratio and reduced physical activity, and increased risk of breast cancer [99, 100, 101]. Similarly, epigenetic modifications have been found to be influenced by environmental exposures in women at high risk of breast cancer relative to healthy controls [12, 90, 102].

While genetic and environmental exposures are known to contribute to breast cancer risk, the magnitude of interaction between these remains undefined for many genes [103, 104, 105, 106, 107]. However, there is evidence that host and environmental factors can modify the penetrance in germline *BRCA1/2* mutation carriers. Increased fat mass and dysmetabolism are recognized to promote breast cancer risk in the general population but there is evidence to indicate a greater impact in *BRCA2* carriers [108], and diet has also been shown to influence *BRCA* penetrance [109]. It has also been shown that smoking for at least 5 years prior to a first pregnancy increases the risk of developing breast cancer in *BRCA1/2* mutation carriers [110]. A recent review outlines the complexity of gene–environment interactions in *BRCA1/2* mutation carriers [111].

Molecular pathological epidemiology studies that integrate data from the clinical records of patients will help to clarify the causal relationships between environmental exposures, pathologic interpretations, molecular aberrations, and disease evolution within the cancerized field.

Biobanks can facilitate such research by implementing protocols to gather information pertaining to lifestyle and environmental exposures, as well as procedures for the collection, management, storage, and distribution of longitudinal microbiota specimens.

### Designating appropriate control cohorts

Collecting a germline genetic profile from buffy coat samples is common practice in DNA sequencing studies. However, it is less common to have matched tissue control samples for comparative transcriptomics, where spatial and temporal effects are significant confounders.

That the field of HN peri‐tumoral tissues may have undergone a molecular insult is an inherent problem in study design. Using cancer‐adjacent tissues to represent a ‘healthy’ control could provide a false representation of baseline gene expression, leading to inaccurate tumor profiles, as well as limiting the opportunity to order tumor‐associated molecular alterations chronologically.

Designation of an appropriate control cohort is dependent on the objective of a study. While cancer‐adjacent samples may have acquired molecular alterations, use of contralateral tissues could prove invaluable when conducting intra‐individual and site‐specific comparisons. Furthermore, pan‐cancer studies have reported that changes in gene expression between paired tumor and adjacent tissues improve the forecast of disease aetiology and patient outcome more than tumor alone [14].

The use of a control cohort of breast tissue from women without breast cancer (nor at known increased risk of breast cancer), such as tissue from reduction mammoplasties, is often preferred. However, their use introduces inherent variability into analyses, particularly when attempting to determine subtle patient‐specific variations. This heterogeneity can be minimized by adopting stringent pre‐defined inclusion criteria, such as demographic (age, ethnicity, gender) or clinical (age of menarche, menopausal status, parity, BMI) and lifestyle features (smoking, contraceptive use), matching those of the breast cancer cohort.

It is important to elucidate alterations within the cancerized field as well as their clinical implications because it is these cancer‐adjacent tissues that will remain unresected in commonly accepted guidelines and in breast conservation therapy. Researchers need to assess the strengths and weaknesses of each candidate set of controls, to make an informed decision and delineate appropriate baseline cohorts. In addition, they also need to have access to specimens or data with complete clinical and surgical information from independent cohorts for validation.

## Exploiting the peri‐tumoral field for patient benefit

Natural processes associated with ageing, such as methylation and a lifetime exposure to endogenous and exogenous factors, cause genomic changes that resemble those of early cancer [9, 71, 112, 113]. However, treating the whole body as a cancerized field has limited clinical utility. Similarly, germline breast cancer susceptibility gene variants, such as *BRCA1* and *BRCA2*, are not considered to contribute towards the traditional definition of a cancerized field [11].

While the cancerized field represents a promising therapeutic target, it is important to acknowledge that presence of the field alone will not determine progression to cancer or propensity to metastasize. To avoid the pitfalls of overdiagnosis and overtreatment, we need to understand the underlying mechanism and molecular events and be able to differentiate between changes that drive predisposed lineages further down the tumorigenic evolutionary pathway and those changes that have minimal cancerization effect.

Currently, invasive procedures, such as colonoscopy and cervical cytology, tend to be employed for the early detection of cancer. Non‐invasive detection strategies usually rely on detecting cancer‐specific biomarkers, which are developed for their ability to differentiate between tumor and normal tissues. Many of these strategies detect the presence of late‐stage cancer or metastasis, with few able to detect early‐stage disease [114, 115, 116].

Two major liquid biopsy studies, CancerSEEK and GRAIL, support the premise that circulating tumor DNA is a promising biomarker for diagnostic screening for sporadic cancer. CancerSEEK analysed mutated DNA and eight standard protein biomarkers in 1005 patients previously diagnosed with stage I–III colorectal, breast, gastric, liver, oesophageal, ovarian, and pancreatic cancers [114]. GRAIL applied targeted whole‐genome bisulphite sequencing of plasma DNA to identify distinct methylation patterns associated with specific cancers to detect a number of those cancers early and simultaneously provide information about the organ of origin [116]. This prospective multi‐center case–control study comprised 6689 individuals, split one third with cancer and two thirds without cancer, representing more than 50 primary cancer types across all clinical stages. While both studies exhibit specificities of more than 99%, sensitivity was proven to be dependent on stage (CancerSEEK, ~40% in stage I disease to approximately 80% in stage III disease; GRAIL, ~18% in stage I disease to ~91% in stage IV disease).

Circulating tumor DNA has been shown to recapitulate a subset of mutational signatures identified in the primary tumor and to have good correlation with tumor burden in solid tumors [117, 118, 119, 120, 121, 122]. Breast cancer studies have reported elevated circulating tumor DNA burden to be a strong predictive marker of disease progression, decreased progression‐free and overall survival, and poor response to treatment [118, 123, 124, 125, 126]. However, almost no studies into solid tumors have attempted to detect distinct cancer‐adjacent profiles in liquid biopsies.

Wu *et al* conducted a study on 27 patients with head and neck squamous carcinoma [12]. The authors observed mutations unique to cancer‐adjacent tissues in post‐operative liquid biopsies from blood and saliva samples from a subset of patients. Moreover, the study reported that integrating data on both cancer‐adjacent‐specific mutations and tumor‐specific mutations increased the sensitivity of post‐operative monitoring to predict relapse. These observations raise the question of whether treatment and predictive models could benefit from insights gained from distinct signatures originating from the cancerized field.

Studies indicating the importance of HN cancer‐adjacent tissues in the context of their ability to prognosticate outcome and response to treatment highlight the requirement for further investigations [12, 57, 63]. Understanding the intricacies of field cancerization and developing predictive biomarkers that exploit the characteristics of this cancerized field could help to improve prognostic and therapeutic determinations and inform clinical decision‐making.

It is important to appreciate that field cancerization is dynamic and influenced by a range of genetic and environmental factors over time. As such, longitudinal clinical observation of breast cancer patients would enable clinicians to monitor trends in blood biomarker profiles and determine when the patient's risk of recurrence changes. Clinical management of the patient is thus enhanced by introducing more information to guide the level of surveillance.

Diagnostic tissue biopsies serve to provide a snapshot of the tumor and surrounding tissues at a given point in time. Repeated invasive biopsies are not a feasible solution to monitoring patient disease trajectory, whereas liquid biopsies have potential as clinical modalities able to yield important diagnostic, prognostic, and therapeutic information and to reduce the dependency on invasive procedures and radiological tests.

The demarcation of surgical margins is not consistent between patients. Molecular signatures developed to detect high‐risk peri‐tumoral tissues could be used in conjunction with morphological examinations to define excision margins and determine the completeness of excision in breast conservation therapy. Evidence of clear pathological and molecular margins could indicate decreased risk of recurrence, thereby sparing patients additional radiation therapy. Similarly, evidence of a field biomarker profile associated with increased risk of cancer recurrence could distinguish patients requiring increased surveillance or more aggressive treatment at the time of surgical intervention. As such, it is important for cancerized field‐related concepts to be recorded as a part of medical health records.

## Conclusion

Continued technological advances will undoubtedly have significant repercussions in elucidating the cancerized field. This may result in a paradigmatic shift from histopathological‐driven therapeutics to a holistic evaluation of clinical and molecular characteristics to complement current diagnostic techniques and help inform clinical decision‐making.

## Author contributions statement

EG and CC conceptualized the manuscript. EG, GJT and LJJ wrote the manuscript. EG, GJT, CC, HR‐A and LJ reviewed and edited the manuscript. EG, HR‐A, GJT and CC made revisions.
